# Color‐Tunable and Stable Copper Iodide Cluster Scintillators for Efficient X‐Ray Imaging

**DOI:** 10.1002/advs.202205526

**Published:** 2022-12-03

**Authors:** Wenjing Zhao, Yanze Wang, Yuanyuan Guo, Yung Doug Suh, Xiaowang Liu

**Affiliations:** ^1^ Frontiers Science Center for Flexible Electronics (FSCFE) MIIT Key Laboratory of Flexible Electronics (KLOFE) Shaanxi Key Laboratory of Flexible Electronics Xi'an Key Laboratory of Flexible Electronics Xi'an Key Laboratory of Biomedical Materials & Engineering Xi'an Institute of Flexible Electronics Institute of Flexible Electronics (IFE) Northwestern Polytechnical University Xi'an 710072 China; ^2^ Department of Chemistry and School of Energy and Chemical Engineering UNIST Ulsan 44919 Korea; ^3^ Key laboratory of Flexible Electronics of Zhejiang Provience Ningbo Institute of Northwestern Polytechnical University 218 Qingyi Road Ningbo 315103 China

**Keywords:** copper iodide cluster, radioluminescence, scintillator, tunable bandgap, X‐ray imaging

## Abstract

The search for color‐tunable, efficient, and robust scintillators plays a vital role in the development of modern X‐ray radiography. The radioluminescence tuning of copper iodide cluster scintillators in the entire visible region by bandgap engineering is herein reported. The bandgap engineering benefits from the fact that the conduction band minimum and valence band maximum of copper iodide cluster crystals are contributed by atomic orbitals from the inorganic core and organic ligand components, respectively. In addition to high scintillation performance, the as‐prepared crystalline copper iodide cluster solids exhibit remarkable resistance toward both moisture and X‐ray irradiation. These features allow copper iodide cluster scintillators to show particular attractiveness for low‐dose X‐ray radiography with a detection limit of 55  nGy s^−1^, a value ≈100 times lower than a standard dosage for X‐ray examinations. The results suggest that optimizing both inorganic core and organic ligand for the building blocks of metal halide cluster crystals may provide new opportunities for a new generation of high‐performance scintillation materials.

## Graphical abstract

The radioluminescence tuning of copper iodide cluster scintillators in the entire visible region by bandgap engineering through the choice of organic ligand is reported. The optimized crystalline copper iodide cluster solids show high scintillation performance and remarkable resistance toward moisture and X‐ray irradiation, enabling attractive application for low‐dose X‐ray radiography.

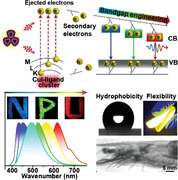

## 1 Introduction

Scintillators with the ability to convert ionizing radiation into low‐energy photons are a crucial component in nuclear detection, industrial nondestructive inspection, and digital radiography.^[^ ^1^, ^2^, ^3^, ^4^, ^5^, ^6^ ^]^ Conventional bulk X‐ray scintillating single crystals, such as CsI:Tl, CdWO_4_ (CWO), and YAlO_3_:Ce (YAP:Ce) are mainly synthesized by the high‐temperature Czochralski strategy.^[^ ^7^, ^8^, ^9^ ^]^ The scintillators permit high X‐ray absorption coefficients because of the heavy constituent atoms and show the promise to produce strong emissions by efficiently converting the high energy of incident X‐ray photons.^[^ ^10^, ^11^, ^12^, ^13^ ^]^ However, conventional scintillators often suffer from hygroscopicity due to the existence of strong ionic bonds.^[^ ^14^ ^]^ In addition, luminescent ion‐based scintillation enables non‐tunable emission color because the luminescence of dopants, especially lanthanides, is inert to the host lattices in most cases due to the shielding effect of the 4f electrons by the completely filled 5s^2^ and 5p^6^ sub‐shells.^[^ ^15^, ^16^, ^17^ ^]^ The emerged lead halide perovskite nanoscintillators with size‐/composite‐dependent bandgap characteristics add the feasibility to the production of multicolor radioluminescence (RL) covering the entire visible region, largely expanding the tools for multicolor radiation detection.^[^ ^1^, ^18^, ^19^ ^]^ In addition, chiral lead halide perovskites emit circularly polarized radioluminescence under irradiation of X‐rays, emerging as a new type of scintillators for improved X‐ray imaging.^[^ ^20^ ^]^ Despite enormous efforts, developing color‐tunable, eco‐friendly, and water‐resistant scintillation materials with high performance remains a formidable challenge.^[^ ^21^ ^]^

Inspired by the unique optical properties of crystalline Cu–I cluster‐based solids, we envision that structure modulation of such crystals provides a much‐needed solution to the above‐mentioned issue.^[^ ^22^, ^23^, ^24^ ^]^ First, heavy atoms of Cu and I in the inorganic (CuI)*_x_* motif in the building blocks enable exceptional X‐ray absorption capability. Moreover, the diversity of the choice of organic ligand allows for bandgap modulation of the resultant crystalline Cu–I cluster structures.^[^ ^25^, ^26^ ^]^ This fact provides flexibility in the generation of tunable RL because both RL and photoluminescence (PL) in semiconductor‐based scintillators are generated by direct recombination of electrons and holes at the edges of the bandgap.^[^ ^27^, ^28^, ^29^ ^]^ Furthermore, crystalline Cu–I cluster structures with high‐efficiency luminescence permit strong RL production because there is likely a linear dependence of light yield for a scintillator on its PL quantum efficiency.^[^ ^30^, ^31^ ^]^ In addition, either intercluster or intracluster interaction engineering has proven effective in enhancing the resistance of Cu–I cluster lattices toward both moisture and X‐rays.^[^ ^32^ ^]^

**Figure 1:**
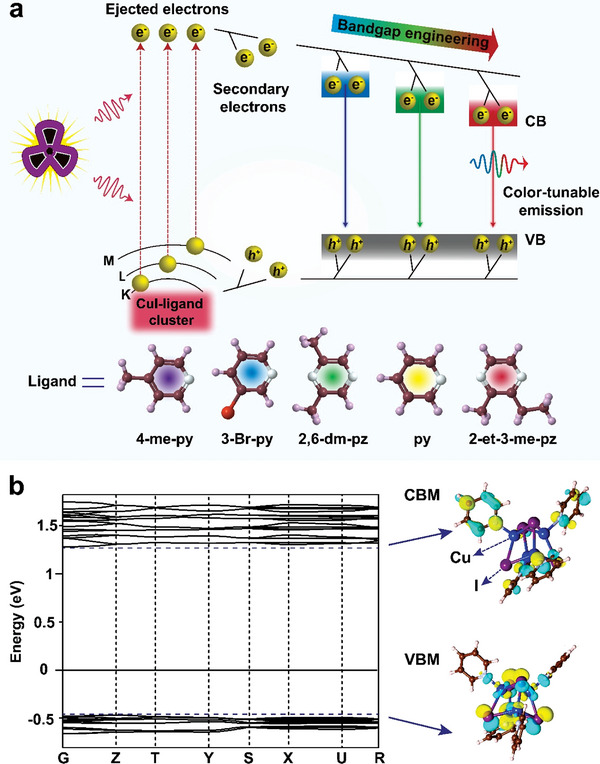
Schematic of scintillation emission tuning of 1D crystalline Cu–I cluster structures. a) Schematic of the proposed scintillation mechanism for crystalline Cu–I cluster structures and scintillation emission tuning by bandgap engineering. Under X‐ray excitation, heavy atoms from the 1D inorganic chains (including Cu and I) play a vital role in absorbing the incident high‐energy rays, allowing for generating mangy energetic primary electrons. The high‐energy primary electrons then lead to the formation of secondary electrons via photoelectric absorption, Compton scattering, and pair formation. The dissipation of the kinetic energy of the secondary electrons during their transport results in many excitons. Efficient radiative recombination of the excitons at tunable band‐edges gives rise to multicolor scintillation emissions. b) Calculated electronic band structure, CBM‐, and VBM‐associated charge densities of CuI(py).

We herein report RL tuning in the entire visible spectral region for Cu–I cluster scintillators in which a Cu–I component and nitrogen‐containing heterocyclic compounds are exploited as an inorganic core and organic ligand, respectively (**Figure** **1**a). The selection of pyridine‐based molecules is due to the fact that the N atom in the ligand can strongly coordinate with Cu(I), allowing for the preparation of stable Cu–I cluster crystals.^[^ ^33^ ^]^ In addition, the diversity of pyridine‐based molecules enables the feasibility of the preparation of a variety of Cu–I cluster crystals with tailored bandgap, permitting the generation of radioluminescence covering the whole visible region. We use (CuI(pyridine))_4_ (Cu(pry)) scintillators that emit intense yellow RL at 565 nm under X‐ray irradiation as a model system to demonstrate their potential for X‐ray detection, enabling a low detection limit of 55 nGy s^−1^, a value ≈100 times lower than the standard dosage for a typical X‐ray medical examination. Combined with their remarkable optical robustness toward both moisture and X‐rays, we present the particular attractiveness of CuI(py) scintillators for flexible and low‐dose X‐ray radiography after being doped into a plastic polymer film.

## 2 Results and Discussion

We began our study by selecting appropriate organic ligand for tuning the bandgap of crystalline Cu–I cluster structures. We first probed the electronic band characteristic of CuI(py) crystals by performing density functional theory calculations. Our results showed that the crystalline CuI(py) solid shows a semiconducting nature with a calculated bandgap of 1.7 eV (Figure 1b). More importantly, calculated conduction band minimum (CBM) and valence band maximum (VBM) of CuI(py) crystals are dominated by atomic orbitals from the inorganic core and organic ligand components (Figure 1b), respectively. These calculated results confirm the feasibility of bandgap engineering of crystalline Cu–I cluster structures through the choice of organic ligand for RL tuning.

**Figure 2:**
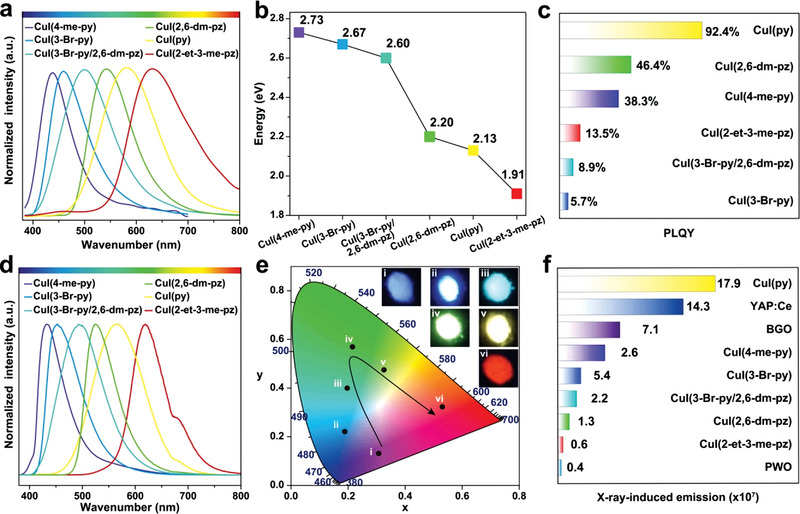
Optical characterization of the as‐prepared crystalline Cu–I cluster scintillators. a) PL profiles under excitation at optimized wavelengths. b,c) Optical bandgap and PLQY of the Cu–I cluster structures. d) RL spectra under X‐ray irradiation (60 kV, 200 µA). e) A CIE chromaticity coordinate diagram of the RL of the crystalline Cu–I cluster scintillators. Insets show the photographs of the as‐prepared Cu–I cluster crystals under irradiation with X‐rays. f) Scintillation performance comparison of Cu–I cluster scintillators and conventional commercially available ones under the same X‐ray irradiation conditions (60 kV, 200 µA).

To validate our hypothesis, we prepared a set of CuI(py‐based ligand) cluster crystals by a wet chemistry method (Figure S1, Supporting Information).^[^ ^34^ ^]^ Pyridine‐based ligands include 4‐methylpyridine (4‐me‐py, C₆H₇N), 3‐pyridyl bromide (3‐Br‐py, C_5_H_4_BrN), 2,6‐dimethylpyrazine (2,6‐dm‐pz, C_6_H_8_N_2_), pyridine (py, C_5_H_5_N), and 2‐ethyl‐3‐methylpyrazine (2‐et‐3‐me‐pz, C_7_H_10_N_2_) (Figure 1a). With prolonged reaction times, the corresponding crystals with varied sizes were precipitated at the bottom of the vials. Successful preparation of the CuI‐pyridine‐based cluster crystals was confirmed by the consistency between the simulated and measured X‐ray diffraction profiles (Figure S2, Supporting Information). As expected, we observed multicolor emissions from the as‐prepared Cu–I cluster crystals upon excitation at 365 nm (Figure S3, Supporting Information). PL profiles show that the emission bands are redshifted from 433 nm for CuI(4‐me‐py) to 564 nm for CuI(py) and to 634 nm for CuI(2‐et‐3‐me‐pz) (**Figure** **2**a). A gradual decrease in the bandgap of the resultant Cu–I cluster structures was further verified by the optical bandgap evaluation based on the corresponding UV–vis absorption profiles (Figure 2b; Figure S4, Supporting Information). Note that the bandgap of the resultant Cu–I cluster crystals can be further adjusted with the use of a binary ligand strategy, as supported by the observation that the resultant products deliver an emission at 493 nm, which is between the emission positions of CuI(3‐Br‐py) (453 nm) and CuI(2,6‐dm‐pz) (526 nm) crystals. We found that the yellow emission at 564 nm for CuI(py) exhibited a quantum yield of 92.4%, the highest one for the emissions in the visible region for the as‐prepared Cu–I cluster structures (Figure 2c). The lifetime of the yellow emission was estimated to be 11.8 µs, indicative of the phosphorescence nature of the yellow emission (Figure S5, Supporting Information).

Realizing bandgap modulation allows for RL tuning spanning the visible spectral region upon X‐ray irradiation. When shifting the excitation source from UV light to X‐rays, we found that the emission profiles are identical to the PL (Figure 2d), suggesting that the luminescence process via a radiative combination of excitons is the same for both PL and RL. The major difference lies in the excitation step. Under high‐energy X‐ray irradiation, heavy atoms of Cu and I account for efficient X‐ray absorption and allow for ejecting mangy energetic primary electrons from their inner shells. The high‐energy primary electrons then lead to secondary electrons via a combination of photoelectric absorption, Compton scattering, and pair formation. The secondary electrons dissipate their kinetic energy by interacting with lattices and other electrons by generating a large number of excitons, which give rise to intense scintillation emissions upon efficient radiative band‐edge recombination.^[^ ^35^, ^36^, ^37^ ^]^

The CIE chromaticity coordinate diagram (Figure 2e) and the corresponding RL photographs (insets, Figure 2e) of the Cu–I cluster scintillators confirm the coverage of the RL in the entire visible region. We further quantitatively compared the scintillation performance of the Cu–I cluster scintillators and the commercially available equivalents under the same excitation conditions (Figure 2f), including YAP:Ce, BGO (Bi_4_Ge_3_O_12_), and PWO (PbWO_4_) single crystals. We found that a 2‐mm thick film comprising Cu–I cluster crystals delivers RL higher than that of a 2‐mm thick PWO single crystal. Note that the strongest RL was observed for CuI(py) cluster structures, ≈1.25, 2.52, and 44.75 times stronger than the emissions for YAP:Ce, BGO, and PWO, respectively. These findings suggest that the scintillation efficiency is likely to show high dependence on the photoluminescence quantum yield (PLQY), consistent with a reported phenomenological model (Equation (1)).^[^ ^30^ ^]^

(1) $$I=\frac{E}{\beta E_{g}}\times S\times Q$$

where *I* is the light yield, *E* the energy of the incident X‐rays, *β* the constant parameter, *E*_g_ the bandgap energy, *S* the host‐to‐emission center energy migration efficiency, and *Q* the quantum efficiency, and the value equals PLQY.

**Figure 3:**
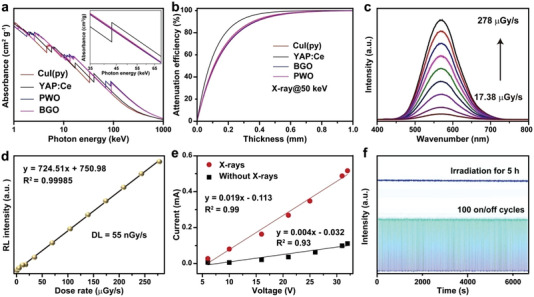
Scintillation characterization of the as‐prepared CuI(py) cluster scintillators. a) X‐ray absorption comparison among CuI(py), YAP:Ce, CWO, and BGO as a function of the irradiation energy. b) The attenuation efficiency of CuI(py), YAP:Ce, CWO, and BGO scintillators as a function of their thickness under irradiation at 50 keV X‐rays. c) The RL spectra of CuI(py) cluster scintillators as a function of X‐ray dose rate in the range of 17.38–278 µGy s^−1^. d) The linear dependence of the RL intensity as a function of dose rate. Note that the detection limit is defined as the dose rate at which the signal‐to‐noise ratio (SNR) equals 3. e) Photocurrent curve for a CuI(py) microcrystal film with and without X‐ray irradiation. f) X‐ray resistance examination of CuI(py) cluster scintillators by continuous X‐ray illumination for 5 h (top) and by on–off repeated irradiation for 100 cycles at a dose rate of 2.85 mGy s^−1^ (bottom).

According to the XCOM web database,^[^ ^38^ ^]^ we used CuI(py) cluster structures as a model system to compare their X‐ray absorption capability with the above‐mentioned inorganic scintillators (**Figure** **3**a and inset). The results showed that the CuI(py) cluster structures exhibit comparable X‐ray absorption to PWO and BGO but weaker absorption than YAP:Ce in the range of 40–60 keV suitable for digital medical radiography.^[^ ^39^ ^]^ The calculated attenuation efficiency of CuI(py) to 50 keV X‐ray photons as a function of the thickness showed the ability of a 2 mm‐thick film to absorb ≈95% of the incident X‐rays (Figure 3b).^[^ ^11^ ^]^ The attenuation effect was much stronger than that of BGO, PWO, and YAP:Ce. We found that the RL intensity of CuI(py) cluster structures showed a linear dependence on the X‐ray dose rate ranging from 17.38 to 278 µGy s^−1^ (Figure 3c). This phenomenon suggests that the luminance process is dominated by radiative recombination of excitons at band‐edges other than by defect recombination.^[^ ^22^ ^]^ The high scintillation performance of the CuI(py) cluster structures enables a detection limit (DL) of 55 nGy s^−1^ based on a three‐time signal‐to‐noise method (Figure 3d),^[^ ^40^, ^41^, ^42^ ^]^ a value ≈100 times lower than the standard dosage for X‐ray examinations (5.5 µGy s^−1^).^[^ ^43^ ^]^ On a separate note, considerable conductivity of a CuI(py) cluster microcrystal film was observed in the dark, and a remarkable increase in the conductivity was then generated when exposing the film to X‐rays at a dose rate of 233 mGy s^−1^ (Figure 3e). These results not only suggest the semiconducting attribute of the CuI(py) cluster crystals but also imply the formation of X‐ray‐induced charge carriers during the transformation of high‐energy X‐rays to low‐energy photons.^[^ ^44^, ^45^, ^46^ ^]^ We further found that the CuI(py) cluster structures exhibit remarkable robustness toward X‐ray irradiation (Figure 3f), as supported by negligible changes in RL intensity under both continuous X‐ray irradiation for 5 h and repeated on–off X‐ray irradiation for 100 cycles at a dose rate of 2.85 mGy s^−1^.

**Figure 4:**
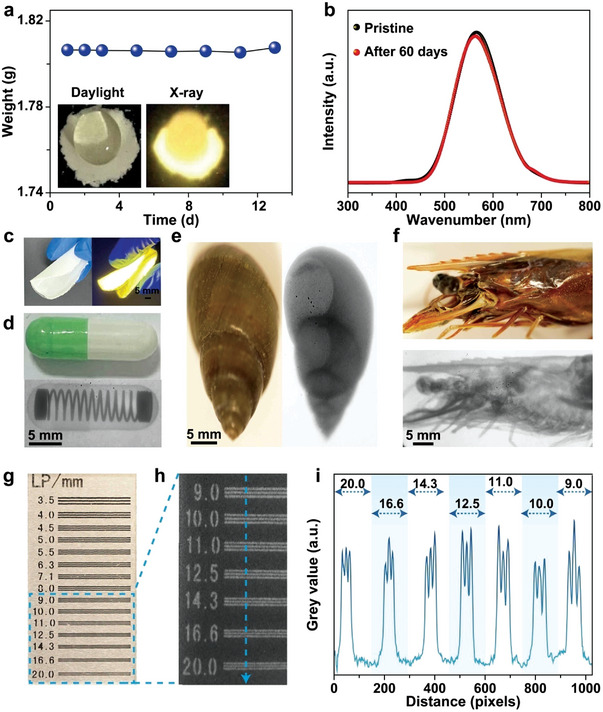
Moisture resistance examination and X‐ray imaging application of the CuI(py) cluster scintillators. a) Weight of CuI(py) cluster structures as a function of time at a relative humidity of 60% (30 °C). Inset showing the CuI(py) cluster scintillator film upon exposure to water in the presence and absence of X‐ray irradiation. b) RL profiles of CuI(py) cluster structures before and after being stored in the air for 60 days. Insets show the corresponding photographs under X‐ray irradiation. c) Photographs of a CuI(py) cluster microparticle‐doped PVA film in daylight and under UV irradiation. d–f) X‐ray images of a capsule containing a metallic spring, a spiral with a helical structure, and a head of a shrimp with different tissue densities. Note that the corresponding photographs were provided in the insets. g,h) The optical and X‐ray photographs of a standard X‐ray resolution pattern plate. i) The grayscale function of the X‐ray photograph ranging from 9.0 to 20.0 lp mm^−1^.

In a further set of experiments, we found that the as‐prepared CuI(py) cluster scintillators show extraordinary moisture resistance. A marginal weight increase was observed after the scintillators were stored at a relative 60% humidity (30 °C) for 13 days (**Figure** **4**a). Furthermore, a strong RL was generated even when the scintillators were directly exposed to water under X‐ray irradiation (inset, Figure 4a). The high moisture stability was likely due to the hydrophobic nature of the CuI(py) cluster structures, as evidenced by a contact angle of 126° at the interface between the CuI(py) cluster structures and a droplet of water (Figure S6, Supporting Information). As expected, no RL degradation was observed for the CuI(py) cluster structures before and after being stored in the air for 60 days without additional protection (Figure 4b).

The ability of CuI(py) cluster scintillators to emit strong RL under X‐ray irradiation, together with their remarkable resistance toward both moisture and X‐ray exposure, allows us to examine their application in low‐dose X‐ray imaging.^[^ ^47^, ^48^, ^49^ ^]^ We first prepared a flexible scintillation film by doping CuI(py) cluster microscintillators into polyvinyl alcohol (PVA) at 10 wt% and found that the resultant film exhibited a strong yellow emission under both UV and X‐ray irradiation (Figure 4c). It is important to note that the as‐prepared film was uniform, and CuI(py) microscintillators were found to disperse homogeneously within the film (Figure S7, Supporting Information). The use of CuI(py) microscintillator‐doped PVA film as a scintillation screen other than CuI(py) microscintillator film was due to the fact that the latter is non‐transparent and shows a strong light scattering effect. We then examined the X‐ray imaging performance of the plastic film by a homemade system (Figure S8, Supporting Information). An X‐ray pattern can be produced after X‐rays penetrate through an object due to the difference in the X‐ray absorption capability of its inner components. The X‐ray pattern is then decoded using a scintillation film of CuI(py) microparticle‐doped PVA to convert the intensity difference in the penetrated X‐rays to varied RL emissions. The resultant RL pattern on the plastic scintillation screen is thereby recorded by a digital camera. Our results show that a metallic spring in a capsule, a helical structure of a spiral, and different tissue densities of the head of a shrimp, can be clearly visualized in the resultant 2D X‐ray photographs (Figure 4d–f). On a separate note, X‐ray imaging of a standard resolution pattern plate suggested a resolution of ≈20.0 line pairs per millimeter (Figure 4g,h) of the as‐built X‐ray imaging system. The generation of the high resolution was then confirmed by a gray value analysis of the light and dark stripes within a gap of a millimeter (Figure 4i). These results suggest the particular attractiveness of Cu–I cluster scintillators for high‐resolution and low‐dose X‐ray radiography.^[^ ^50^, ^51^ ^]^

## 3 Conclusion

In conclusion, we have demonstrated the ease of realization of color tuning of scintillation emissions of Cu–I cluster‐based scintillators by ligand‐assisted bandgap engineering. Our results show that by ligand structure modulation, the scintillation emissions can be tuned to cover the entire visible spectral region. We further presented the attractive application of CuI(py) cluster scintillators for high‐resolution and low‐dose X‐ray radiography due to their outstanding scintillation performance and excellent moisture and X‐ray robustness. Considering the structural diversity in both the inorganic and organic components in clusters, our strategy provides a practical pathway for scintillation optimization of cluster‐derived crystalline structures in terms of efficiency, color output tuning, and lattice robustness.

## 4 Experimental Section

### Materials

4‐me‐py (99%) and 3‐Br‐py (98%) were purchased from the Beijing Innochem Science & Technology Co., Ltd. 2,6‐dm‐pz (99%) and 2‐et‐3‐me‐pz (99%) were obtained from the Shanghai Haohong Biomedical Technology Co., Ltd. PY (99%), PVA (M.W. ≈ 1000) were acquired from the Shanghai Aladdin Biochemical Technology Co., Ltd. Ethanol, acetone, and acetonitrile were provided by Sinopharm Chemical Reagent Co., Ltd. Potassium iodide (KI, 99%) and copper(I) iodide (CuI, 99%) were purchased from Alfa Aesar.

### Synthesis of Cu–I cluster scintillators

A saturated KI solution was prepared in advance for subsequent procedures. CuI (1 mmol) and organic ligand (1 mmol) were then dissolved in the saturated KI solution and organic solvent to form homogeneous solution, respectively. After then, the as prepared KI‐saturated CuI solution (2 mL), acetonitrile (2 mL), and the ligand solution (2 mL) were subsequently added into a 20‐mL vial in the absence of stirring. The mixture was allowed to react at room temperature for 2 days. Upon the completion of the reaction, corresponding products were collected by filtration and dried in the oven at 60 °C.

### Preparation of a CuI(py) Microscintillator‐Doped Plastic Film

CuI(py) cluster scintillators (10 wt%) were first added into a mixture of PVA (5 g) and H_2_O (45 mL) to enable the formation of a homogeneous dispersion under sonication. Then, the homogeneous solution was transferred to a round mold with a diameter of 6 cm made from polytetrafluoroethylene, and the mold was allowed to heat at 40 °C for 2 h to afford a flexible scintillation film.

### Instrumentation

Powder X‐ray diffraction was measured by a Bruker D8 Advance X‐ray diffractometer with Cu K*α* radiation. PL and luminescence decay were obtained on FSL‐1000 (Edinburgh Instruments Ltd.). PLQY was measured by FSL‐1000 (Edinburgh Instruments Ltd.) equipped with an integrated sphere. RL was recorded by an Edinburgh FS5 fluorescence spectrophotometer equipped with an external miniature X‐ray source (AMPEK, Inc.). X‐ray imaging was performed by a homemade setup with the use of an external miniature X‐ray tube, a CuI(py) cluster structure‐doped film, and a digital camera as excitation, scintillation screen, and detector, respectively.

## Conflict of Interest

The authors declare no conflict of interest.

## Supporting information

Supporting Information

## Data Availability

The data that support the findings of this study are available in the supplementary material of this article.
