# Supplementary material for: Color‐Tunable and Stable Copper Iodide Cluster Scintillators for Efficient X‐Ray Imaging
Source: Adv Sci (Weinh). 2022 Dec 3;10(5):2205526. doi: 10.1002/advs.202205526 (PMC9929111; doi:10.1002/advs.202205526)
Supplement: Supplementary file 1 — Supporting Information [file ADVS-10-2205526-s001.pdf]

Supporting Information

Color-Tunable and Stable Copper-Iodide Cluster Scintillators for Efficient X-ray Imaging

Wenjing Zhao, Yanze Wang, Yuanyuan Guo, Yung Doug Suh, Xiaowang Liu, and Wei Huang**

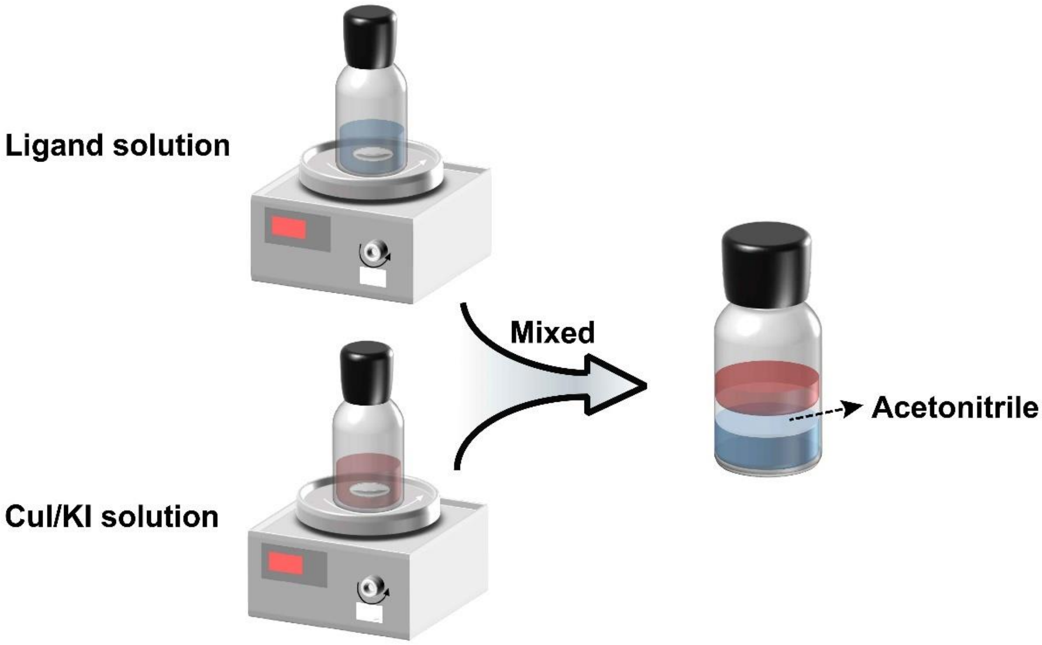

Figure S1. Schematic diagram of the synthesis of CuI(py-based ligand) cluster crystals *via* a wet chemistry method.

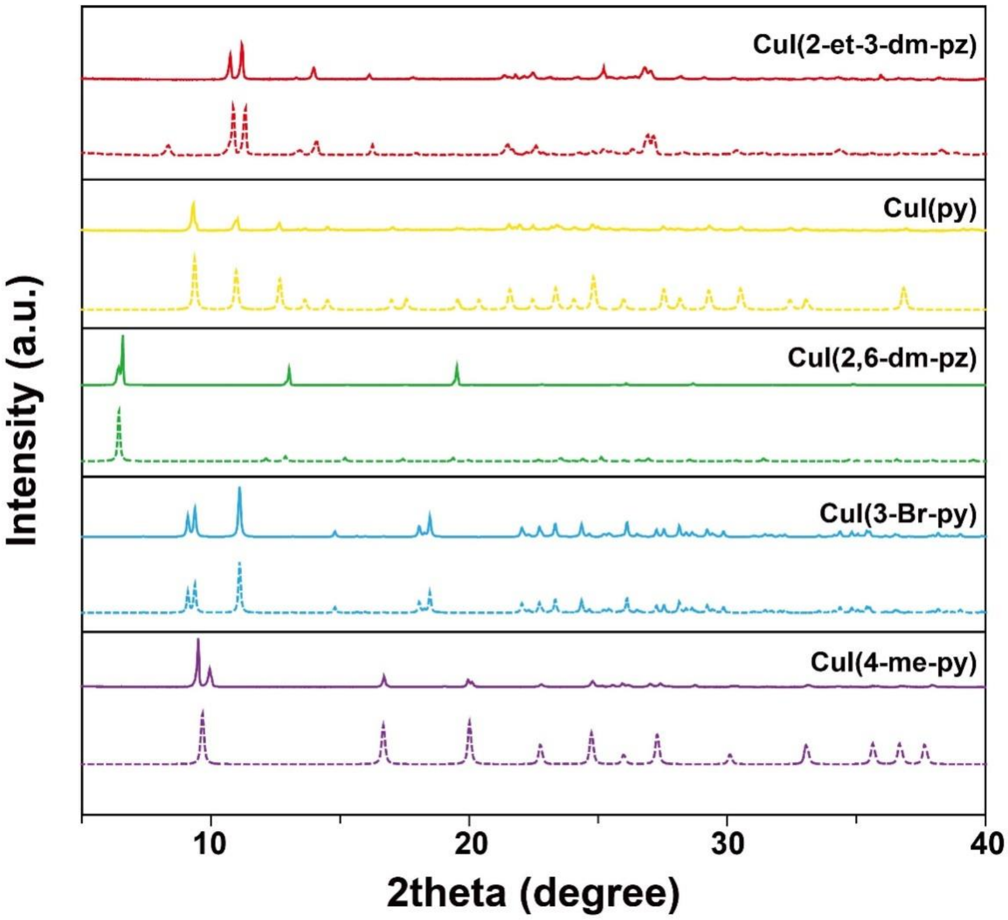

Figure S2. Comparision of the measured (solid line) and simulated (dash line) X-ray diffraction profiles of the as-prepared CuI(py-based ligand) cluster crystals, including CuI(4-me-py), CuI(3-Br-py), CuI(2,6-dm-pz), CuI(py), and CuI(2-et-3-dm-pz).

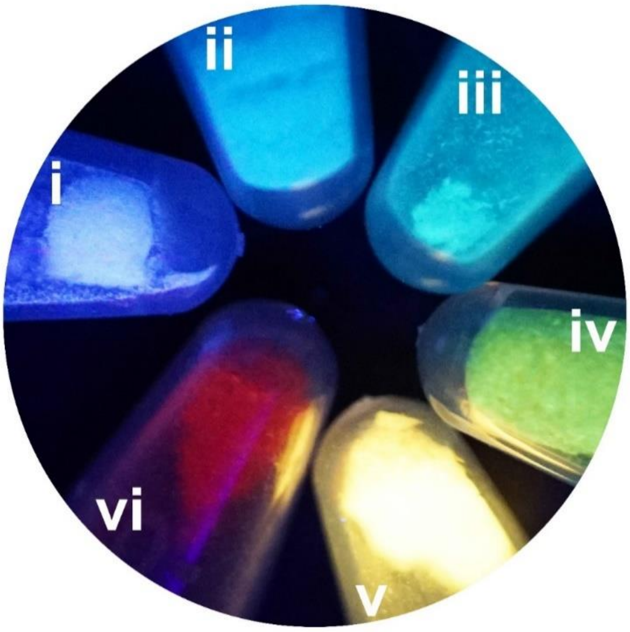

Figure S3. Photographs of the as-prepared CuI(py-based ligand) cluster crystals under irradiation at 365 nm. (i) CuI(4-me-py); (ii) CuI(3-Br-py); (iii) CuI(3-Br-py/2,6-dm-pz); (iv) CuI(2,6-dm-pz); (v) CuI(py); (vi) CuI(2-et-3-dm-pz).

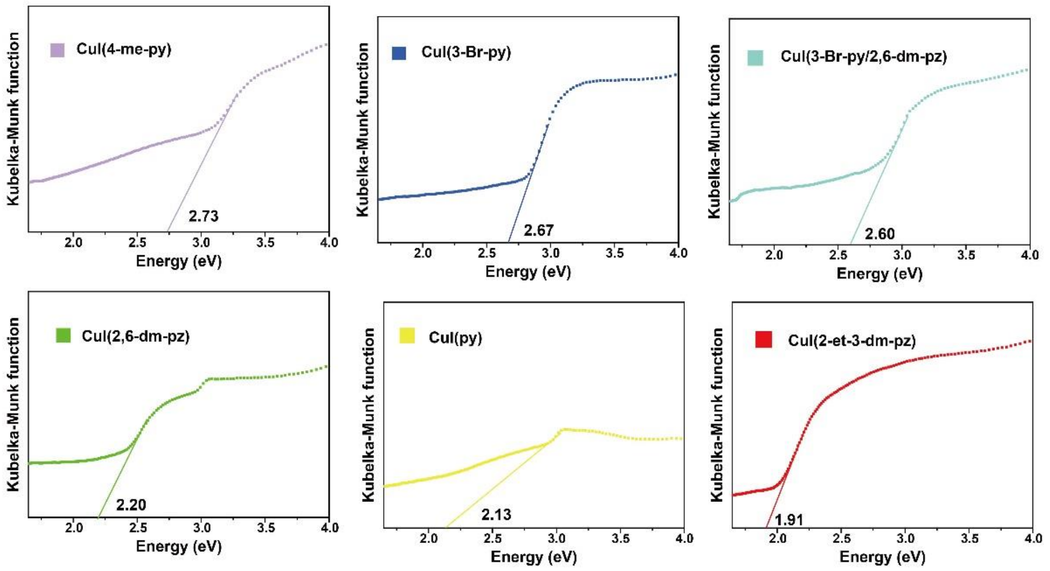

Figure S4. Optical bandgap evaluation of the as-prepared CuI(py-based ligand) cluster crystals.

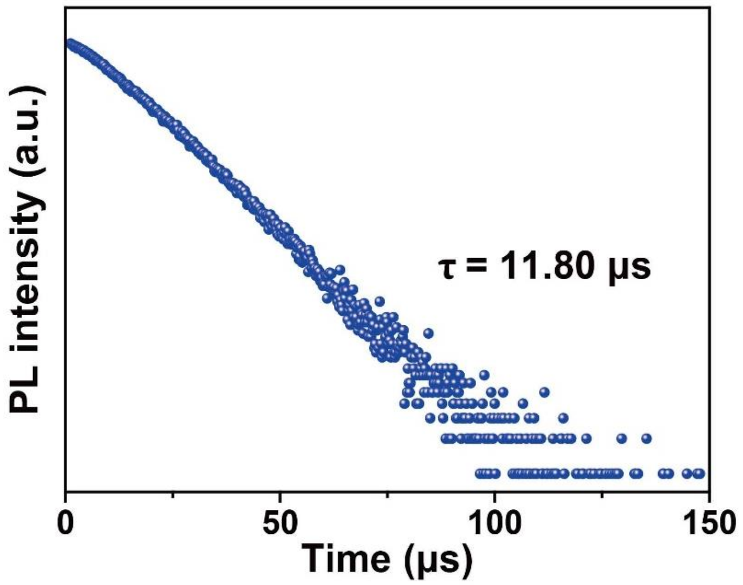

Figure S5. Time-resolved PL decay curves (at 564 nm) of CuI(py) upon exciation at 370 nm.

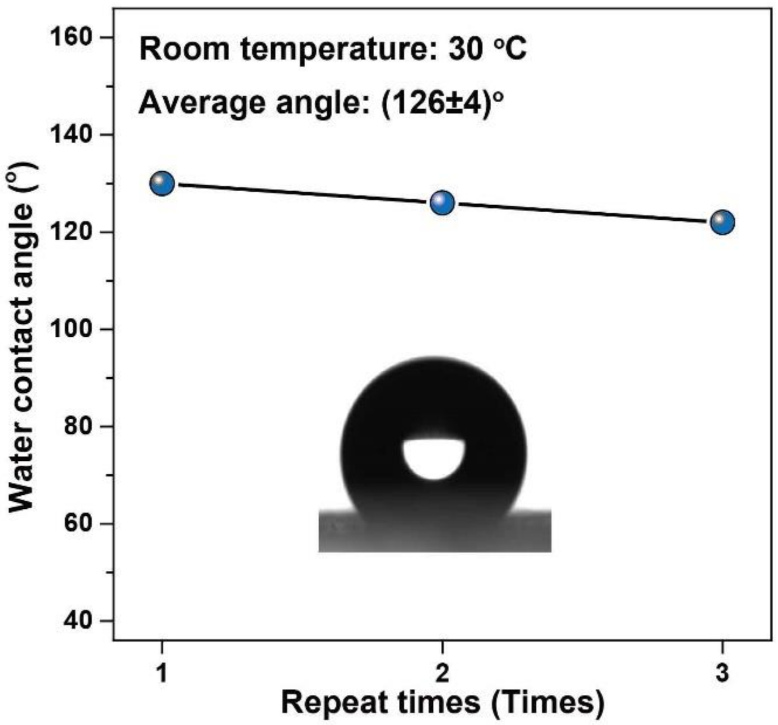

Figure S6. Hydrophobic evaluation of CuI(py) microparticles.

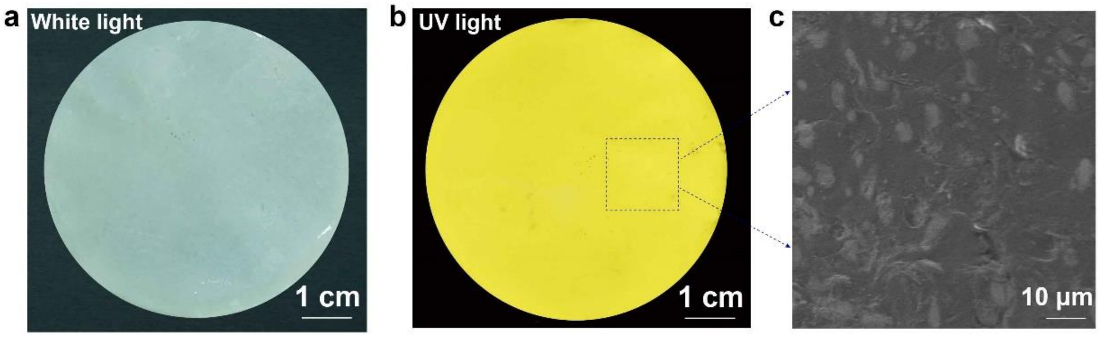

Figure S7. (a and b) Photographs of the CuI(py) microscintillator-doped plastic film with and without UV irradiation. (c) SEM image of the CuI(py) microscintillator-doped plastic film.

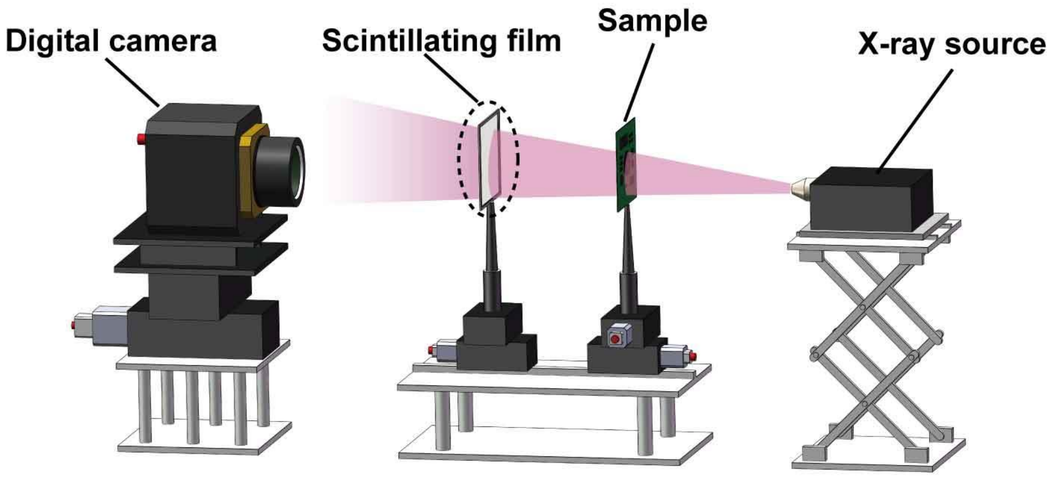

Figure S8. Schematic illustration of a homemade setup used for X-ray imaging.
